# Transnasal Endoscopic Approach for Osteoid Osteoma of the Odontoid Process in a Child: Technical Note and Systematic Review of the Literature

**DOI:** 10.3390/brainsci12070916

**Published:** 2022-07-13

**Authors:** Giuseppe Roberto Giammalva, Letizia Dell’Aglio, Brando Guarrera, Valentina Baro, Leonardo Calvanese, Gloria Schiavo, Giulia Mantovani, Valentina Rinaldi, Domenico Gerardo Iacopino, Francesco Causin, Piero Nicolai, Marco Ferrari, Luca Denaro

**Affiliations:** 1 Unit of Neurosurgery, Post Graduate Residency Program in Neurosurgery, Department of Biomedicine, Neuroscience and Advanced Diagnostics, University of Palermo, 90100 Palermo, Italy; gerardo.iacopino@gmail.com; 2 Academic Neurosurgery, Department of Neurosciences DNS, University of Padua, 35128 Padua, Italy; letizia.dellaglio@aopd.veneto.it (L.D.); brando.guarrera@aopd.veneto.it (B.G.); valentina.baro@aopd.veneto.it (V.B.); luca.denaro@unipd.it (L.D.); 3 Unit of Otorhinolaryngology—Head and Neck Surgery, Department of Neurosciences DNS, “Azienda Ospedale Università di Padova”, University of Padua, 35128 Padua, Italy; leonardo.calvanese@aopd.veneto.it (L.C.); gloria.schiavo@aopd.veneto.it (G.S.); piero.nicolai@unipd.it (P.N.); marco.ferrari@unipa.it (M.F.); 4 Unit of Anesthesia and Intensive Care, Integrated Didactic-Scientific Healtcare Department of Surgery (DIDAS Chirurgia), “Azienda Ospedale Università di Padova”, University of Padua, 35128 Padua, Italy; giulia.mantovani@aopd.veneto.it (G.M.); valentina.rinaldi@aopd.veneto.it (V.R.); 5 Unit of Neuroradiology, Department of Neurosciences DNS, University of Padua, 35128 Padua, Italy; francesco.causin@aopd.veneto.it; 6 Guided Therapeutics (GTx) Program International Scholarship, University Health Network (UHN), Toronto, ON M5G 2C4, Canada; 7 Technology for Health (Ph.D. Program), Department of Information Engineering, University of Brescia, 25123 Brescia, Italy

**Keywords:** osteoid osteoma, odontoid, transnasal approach, endoscopy, pediatric neurosurgery

## Abstract

Osteoid osteoma (OO) is a primary benign tumor that accounts for up to 3% of all bone tumors. The cervical spine is less affected by OOs, and very few cases of C2 OOs have been reported in the literature, both in adults and children. Surgery may be required in case of functional torticollis, stiffness, and reduced range of motion (ROM) due to cervical OOs refractory to medical therapy. Several posterior and anterior surgical techniques have been described to remove C2 OOs. In particular, anterior approaches to the cervical spine represent the most used surgical route for treating C2 OOs. We describe the first case of OO of the odontoid process removed through a transnasal endoscopic approach with the aid of neuronavigation in a 6-year-old child. No intraoperative complications occurred, and the post-operative course was uneventful. The patient had immediate relief of neck pain and remained pain-free throughout the follow-up period, with complete functional recovery of the neck range of motion (ROM). In this case, based on the favorable anatomy, the transnasal endoscopic approach represented a valuable strategy for the complete removal of an anterior C2 OO without the need for further vertebral fixation since the preservation of ligaments and paravertebral soft tissue.

## 1. Introduction

Osteoid osteoma (OO) is a primary benign tumor that accounts for up to 3% of all bone tumors [1,2]. It usually consists of an osteolytic lesion made of a central nidus surrounded by sclerotic margins [2]. The pathological examination of OO commonly shows irregularly calcified osteoid trabeculae and a vascularized stroma [2]. Despite the higher incidence in long bones, up to 10% of OOs occur in the spine, particularly in lumbar vertebrae, representing one of the rarest tumors of the cervical spine [1,3,4]. In fact, very few cases of C2 OOs have been reported in the literature thus far.

Age at the diagnosis ranges between 5 and 25 years, with anecdotal cases reported during infancy [2]. Local pain is often the predominant symptom, which markedly increases during the night due to nidus nociceptive stimulation by the release of prostaglandins [1,5]. Despite the good response to salicylates and nonsteroidal anti-inflammatory drugs (NSAIDs), surgical removal of OOs is advocated in cases of drug-refractory pain and/or associated functional limitations. In particular, surgery may be required in case of acquired torticollis, neck stiffness, and reduced range of motion (ROM) due to cervical OOs [1]. 

Several surgical approaches and techniques have been proposed to treat C2 OOs. Based upon tumor localization and relationships with neighboring structures, anterior and posterior approach have been used, with or without cervical fixation, to obtain complete OOs removal.

As an addition to the spectrum of surgical routes, we describe the first case of C2 OO removed through a transnasal endoscopic approach in a child. The case is herein detailed, and the surgical technique is described, supported by a systematic review of literature about C2 OOs.

## 2. Materials and Methods

### 2.1. Technical Note

#### 2.1.1. Clinical Presentation, Diagnosis, and Initial Conservative Therapy

**Figure 1:**
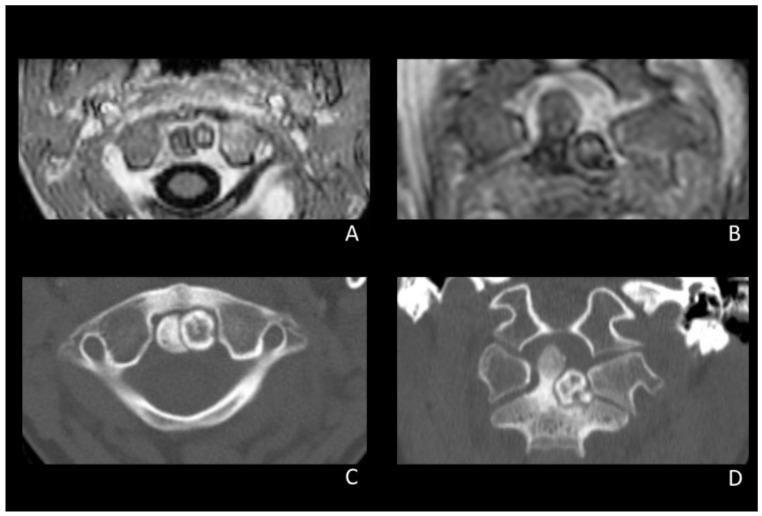
Pre-operative MRI (**A**,**B**) and CT scan (**C**,**D**) showing the calcified lesion on the left portion of the odontoid process adjacent to the left C1-C2 joint.

A 6-year-old girl was referred to the outpatient clinic of our Institution complaining of persistent neck pain for 8 months, which worsened at night, and was unresponsive to acetaminophen. Three months after pain onset, the patient presented progressive functional limitation in lateral bending and rotation of the head. Neurological examination was otherwise unremarkable, and blood exams were normal at the time of the first visit. Contrast Magnetic Resonance Imaging (MRI) revealed a rounded formation of about 7–8 mm axial diameter located on the left portion of the odontoid process. The lesion appeared adherent to the base of the odontoid process and axis body, adjacent to the left C1-C2 joint, and was characterized by a calcific-like appearance. The surrounding prevertebral and paravertebral tissues, together with the adjacent trabecular bone, were edematous (Figure 1A,B).

The patient underwent a cervical spine CT scan, which revealed a thin hypodense border between the outer lesion boundary and the C2 bone. The base of the odontoid process and part of the C2 body looked thickened as they had been imprinted by the lesion growth (Figure 1B,C).

According to the imaging, a differential diagnosis including primary bone lesions such as osteochondroma, inflammatory arthritis with associated synovial calcifications, and osteoid osteoma was hypothesized. Autoimmune and inflammatory markers were negative, and rheumatologic evaluation was inconclusive. 

Due to worsening functional limitation, the patient initially undertook NSAID therapy and underwent physical therapy. A few months later, since the progressive worsening of all symptoms irrespective of conservative therapies, the patient and her parents were proposed to undergo surgical removal of the lesion. 

**Figure 2:**
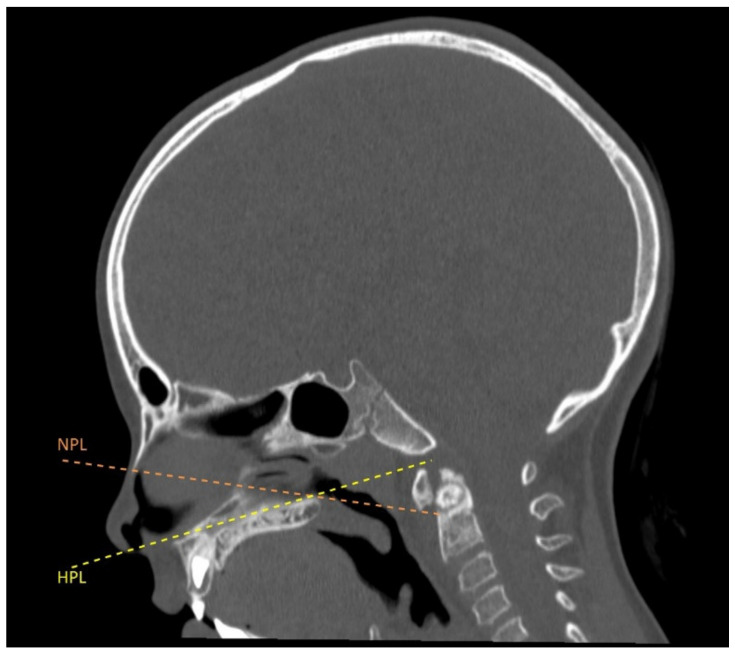
Surgical corridor through the favorable nasopalatine angle (NPL: Nasopalatine line or Kassam line; HPL: hard palate line).

Considering the need for surgery, she was admitted to our Institution. On admission, the patient complained of cervical pain with marked neck stiffness, acquired left torticollis, and severely reduced ROM; no neurological impairments were noticed. The case was discussed by a multidisciplinary team of neurosurgeons and otolaryngologists, and the endoscopic transnasal route was selected as the most convenient surgical approach in view of the favorable trajectory towards the lesion and minimal tissue manipulation needed to reach and excise the lesion (Figure 2). 

#### 2.1.2. Surgical Procedure

**Figure 3:**
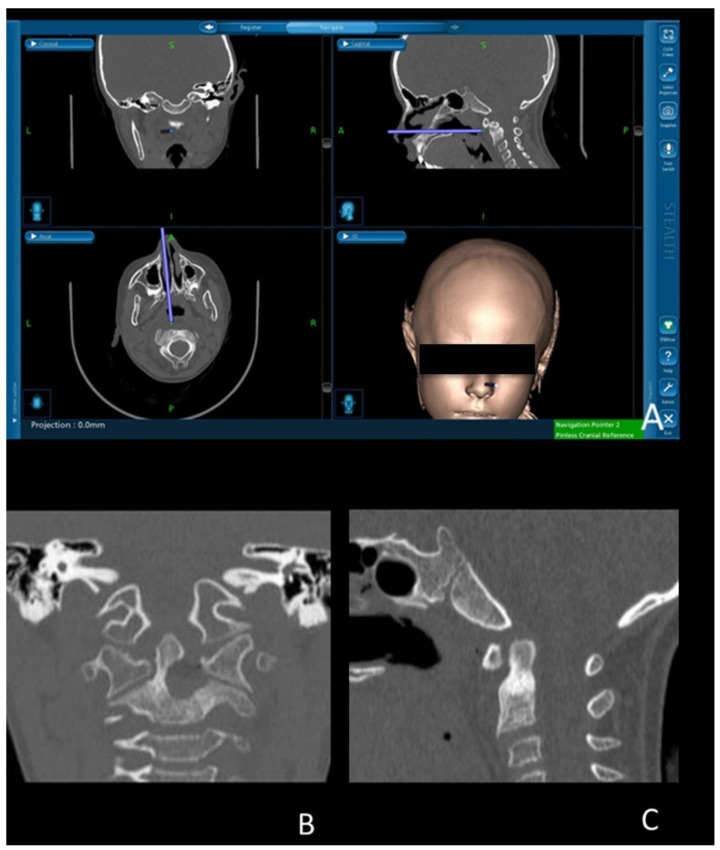
(**A**) Intraoperative neuronavigation during lesion removal; (**B**,**C**) Post-operative cervical CT scan showing the complete removal of C2 osteoid osteoma through transnasal endoscopic approach, with satisfactory sparing of the healthy odontoid process. **Purple line**: intraoperative surgical trajectory.

The surgical procedure was performed through a 2-surgeon, 4-hand technique by otolaryngologists and neurosurgeons, with the patient under general anesthesia. The transnasal endoscopic approach was aided by intraoperative neuronavigation (StealthStation S7, Medtronic Inc., Minneapolis, MN, USA) (Figure 3a). The first phase of surgery consisted of creating the surgical corridor towards the posterior nasopharyngeal wall, which was achieved by removing the tails of inferior turbinates and performing a posterior-inferior septectomy. Then, a myomucosal flap, including the nasopharyngeal mucosa and cranial insertion of the longus capitis muscle, was harvested through an inverted-U-shaped incision along the lateral nasopharyngeal recesses, lateral aspects of the nasopharyngeal vault, and upper choanal border. The flap was dissected off the clivus, anterior atlanto-occipital membrane, anterior arch of the atlas, and anterior atlanto-axial ligament and stored in the oropharynx with a transorally-placed hemostat. The lower half of the left and midline portion of the anterior arch of the atlas was drilled. The lesion was found immediately posterior to the anterior arch of the atlas and was not cleavable from the odontoid process and C2 body. The lesion was cavitated through its anterior wall by using a 15° diamond burr tilted downward, and the outermost cranial, lateral, and posterior portions were dissected, removed, and sent for pathological examination. Since the lack of an appreciable boundary between the OO and axis, the medial and inferior walls were drilled until the identification of macroscopically normal bone (Figure 3A–C). Intraoperative navigation was used to check the boundaries of the drilled cavity to make sure that the OO was completely removed. The drilled cavity was filled with a graft of prevertebral musculature, gelatin sponge, and fibrin glue. Finally, the myomucosal nasopharyngeal flap was positioned in its native situation and sutured to the mucosa of the sphenoethmoidal recesses, and nasal packing was placed. Total blood loss accounted for 350 mL, mostly due to bone drilling and manipulation. Intraoperative video recording of the surgical procedure may be foud in Supplementary Materials (Video S1). 

#### 2.1.3. Post-Operative Course and Follow-Up

Since the intraoperative evidence of ligamentous integrity, vertebral fixation was deemed unnecessary. After the surgery, the patient was briefly admitted to the pediatric intensive care unit and then transferred to the ward. The post-operative course was uneventful, and the patient had immediate relief from neck pain. She fed orally from the second post-operative day and was mobilized with a Philadelphia collar on the same day. An early post-operative CT scan of the cervical spine confirmed the total gross removal of the lesion without signs of complications. 

Pathological examination of the lesion confirmed the diagnosis of OO.

Thus, the patient was prescribed to wear a cervical collar for 1 month to facilitate the subsequent postural rehabilitation. During the follow-up period, the patient underwent endoscopic medication under sedation and local anesthesia on the second day, 2 weeks, and 1 month after surgery.

**Figure 4:**
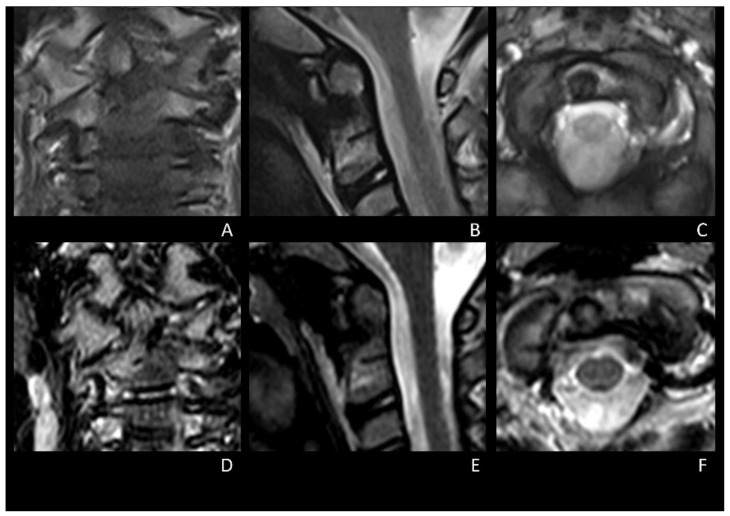
4-months (**A**–**C**) and 6-months (**D**–**F**) post-operative cervical MRI showing progressive and regular bone healing, without indirect signs of cervical instability and no relapse of OO.

The collar was dismissed 1 month after surgery. The patient was symptom-free 3 months after surgery. Post-operative cervical MRI performed 4 and 6 months after surgery showed regular bone healing without indirect signs of vertebral instability or lesion relapse (Figure 4A–F). At the 7-month follow-up, the patient was still symptom-free and had recovered her previous cervical ROM. Considering the lack of pain and radiological signs of vertebral instability, a dynamic cervical X-ray was not deemed necessary; thus, it was avoided to reduce radiation exposure. 

### 2.2. Systematic Review of Literature

To assess the rarity and peculiarity of C2 OOs, a systematic literature review was performed according to Preferred Reporting Items for Systematic Reviews and Meta-Analyses (PRISMA) guidelines [6]. This review was not registered, and the review protocol was not prepared on prospective registers; however, the systematic review was conducted as follows. A literature search was performed in December 2021, querying the PubMed database without backward limits with the following medical subject headings (MeSH): (osteoid osteoma) AND (axis); (osteoid osteoma) AND (odontoid); (osteoid osteoma) AND (C2); (osteoid osteoma) AND (endoscopic) AND (cervical). Reference lists of all publications were also screened. Exclusion criteria were inaccessibility to full text, non-English and non-Italian language, studies not pertinent to C2, studies not pertinent to OOs, and studies lacking relevant clinical data (clinical presentation, type of treatment, surgical technique). Data extracted from each study included the location of C2 OO, type of pre-operative imaging, patients’ symptoms, and type of treatment.

## 3. Results

**Figure 5:**
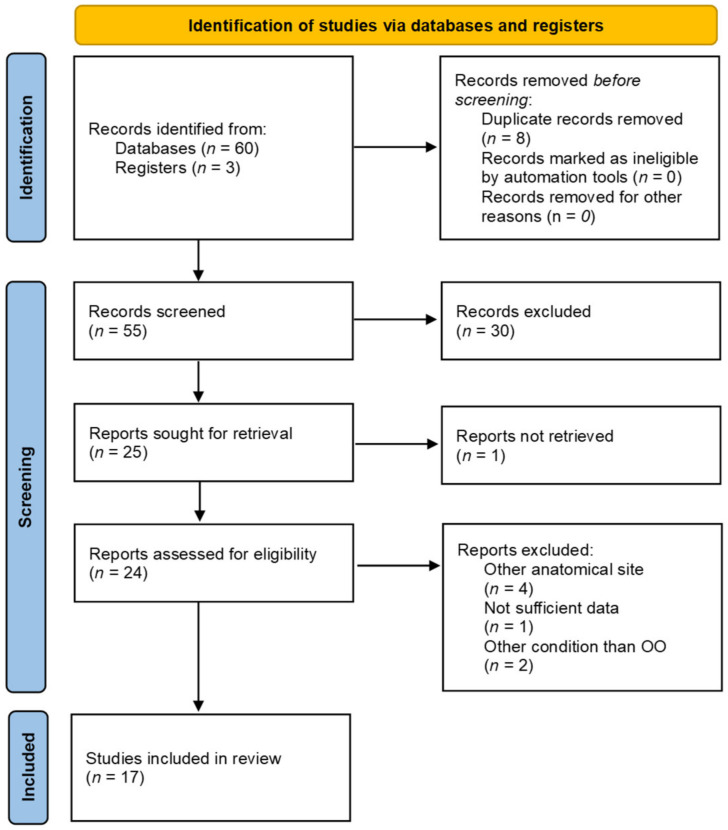
PRISMA flow diagram of the literature review on C2 OOs.

Literature search retrieved the following results: (osteoid osteoma) AND (axis), 19 results; (osteoid osteoma) AND (odontoid), 5 results; (osteoid osteoma) AND (C2), 13 results; (osteoid osteoma) AND (endoscopic) AND (cervical), 23 results. Reference lists of all publications were also screened (3 results). Records screened were 55. Only studies regarding OOs affecting C2 were included. (Figure 5, Table 1).

## 4. Discussion

Vertebral OOs are rare tumors: vertebral bodies are affected in only 10% of OOs [17]. Considering this rarity, the involvement of C2 is exceptional, with only 19 cases of C2 OO reported in the literature thus far, to the best of the authors’ knowledge (Table 1).

Among the included studies, 10 cases of OO involving the C2 body and odontoid process affecting pediatric patients were reported, accounting for 52.6% of all cases. 

Neck pain was the most frequent symptom, which was present in up to 94% of cases and was associated with neck stiffness and functional torticollis in 4 (23.5%) and 5 (29.4%) cases, according to available data, respectively [1,2,3,5,7,8,9,10,11,12,13,14,15,16,17,18,19].

Conservative treatment can be a valuable option in selected cases, both adults and children. In particular, anti-inflammatory treatment with acetaminophen or celecoxib 200mg/day has been proven effective in relieving pain and improving neck ROM for a time ranging between 6 months and 2 years [2,5,7,15].

However, despite the pain relief, only in one case a complete regression of OO after prolonged NSAIDs therapy was reported. Thus, surgical treatment is advised in case of symptomatic OOs refractory to conservative therapy, in case of reduction of pain relief time, and to avoid side effects of prolonged NSAIDs therapy [20,21]. The primary objective of surgery is the removal of the nidus. This can be achieved using different techniques, from en-bloc removal to curettage or microsurgical drilling. In our case, the complete removal of OO was obtained by progressive inside-out drilling of the outer sclerotic shell after cavitating the lesion core through the most easily accessible wall (i.e., the anterior one).

Intraoperative neuronavigation was useful to map the tumor boundaries at the OO-normal bone interface and to confirm the complete removal of the lesion. Until now, in only one other case of C2 OO addressed through the posterior cervical approach, the surgery was aided by neuronavigation [11]. As regards the surgical approach, several surgical techniques have been described to remove C2 OOs. These include both posterior (hemilaminectomy, laminectomy, or laminoplasty), anterior, and antero-lateral approaches [1,2,3,5,7,8,9,10,11,12,13,14,15,16,17,18,19]. The choice of the surgical approach mostly relies upon the OO location. Anterior and antero-lateral approaches to the cervical spine represented the most used surgical route for treating C2 OOs, with 9 out of 15 (60%) surgically treated cases [9,12,13,14,16,17,18,19]. Among these cases, only 3 were managed through an endoscope-assisted approach [16,17,18]. Particularly, 2 patients were operated through an anterior/anterolateral endoscope-assisted approach through the infrayoid trans-fascial route [17,18]. In only one case, a C2 OO was removed through a transoral endoscope-assisted approach [16]. Our case is the first described case of an OO of the odontoid process removed through a transnasal fully endoscopic approach in a child.

Based on the patient’s young age and favorable anatomy, the transnasal corridor was considered the most favorable route for removing the lesion. In fact, the anatomy of this young patient, as seen on the sagittal cranial CT scan, allowed wide surgical exposure of the anterior aspect of cranio-vertebral and atlanto-axial junctions through the transnasal approach [22,23] (Figure 2). Moreover, the transnasal endoscopic approach in such a young patient was deemed preferable by virtue of the low rate of post-surgical infection and pharyngeal wound dehiscence compared with transoral approaches [24]. In addition, early post-operative oral feeding (i.e., from the second post-operative day) was feasible with no need for prolonged nasogastric tube placement. However, the transnasal endoscopic route should be evaluated according to the patient’s singular anatomy, and the surgical approach has to be tailored to the patient’s characteristics and OO location.

In the only similar case in which C2 OO was removed through a transoral endoscope-assisted approach, subsequent stabilization through C1-C2 posterior fixation was required to overcome the atlanto-axial instability given by the complete removal of the C1 anterior arch and the base of the odontoid [16]. Cranio-vertebral and atlanto-axial joint (CVAAJ) stability is a debated topic; in this complex architecture, both bone surfaces and ligaments are thought to determine the stability and motility of these joints [25,26,27]. The risk of instability after CVAAJ surgery, on the one hand, and the determination of bone and soft tissue involvement in maintaining its stability, on the other, are yet debated. However, the contribution of ligaments and periarticular soft tissues in maintaining CVAAJ stability has been pointed out; in particular, preservation of periarticular soft tissue ensures a certain degree of post-operative stability, thus preventing further vertebral fixation [25,26,27]. In keeping with this evidence, the patient did not undergo vertebral fixation since a low risk of post-operative CVAAJ instability was estimated. Considering the patient’s young age and the low risk of CVAAJ, eventual posterior fixation was deferred in case of future evidence of cervical instability.

## 5. Conclusions

OOs of the C2 body and odontoid process are very rare entities that may occur in both adults and children. Provided that the trajectory from the piriform aperture to the target is favorable, the transnasal endoscopic approach represents a valuable strategy for completely removing anterior C2 OOs. Neuronavigation was remarkably useful in locating some tumor boundaries and intraoperatively assessing the complete removal of the OO. Preservation of ligaments and paravertebral soft tissues was paramount to avoid the need for vertebral fixation. 

## Figures and Tables

**Table 1:** Reported case of osteoid osteomas of the axis from systematic review of the literature.

Authors, Year	Pt. Sex, Age	Tumor Localization	Imaging	Symptoms	Treatment
**Children (<18-Year-Old)**					
Neumann, 2007 [2]	F, 14	Right postero-lateral aspect of odontoid process	XR, CT, MRI, bone scintigraphy	Neck pain, occasional tenderness	Medical treatment
Coulier, 2005 [7]	F, 17	Juxta-pedicular, C2 right lateral mass	XR, CT	Neck pain, stiffness, torticollis	Medical treatment
Raskas, 1992 [8]	M, 6	Body of C2	N/A	N/A	Excision (surgical approach not specified)
Bucci, 1989 [9]	M, 7	Left side of odontoid process	XR, CT, scintigraphy	Neck pain, forced position of the head	Transoral macroscopic excision
Kulkarni, 2013 [10]	M, 12	Left C2 lateral mass	CT, MRI	Neck pain	Posterior cervical endoscope-assisted excision
Nagashima, 2010 [11]	F, 12	Left C2 pedicle	XR, CT	Neck pain, reduced ROM	Navigated posterior cervical excision
Eysel, 1994 [1]	M, 15	C2 right lamina	XR, CT	Neck pain, torticollis, reduced ROM	*En bloc* resection and laminoplasty
Molloy, 2002 [12]	M, 15	Posterior aspect of C2 body	XR, CT, MRI, bone scintigraphy	Neck pain, stiffness, torticollis	Antero-lateral cervical macroscopic excision
Al-Balas, 2009 [13]	M, 16	Odontoid process	XR, CT, MRI, bone scintigraphy	Neck pain, occipital headache, tenderness, reduced ROM	Anterior resection of odontoid process and part of C1 anterior arch, C1–C2 anterior fusion
Amirjamshidi, 2010 [14]	M, 17	Left C2 lateral facet	XR, CT, MRI, bone scintigraphy	Neck pain	Hemilaminectomy and *en bloc* excision
**Adults (>=18-year-old)**					
Qiao, 2014 [5]	M, 18	Left lateral aspect of odontoid process	XR, CT	Neck pain, reduced ROM, mild kyphosis	Medical treatment
Aslan, 2015 [15]	F, 47	Odontoid process	CT, MRI	Neck pain	Medical treatment
Ameri, 2019 [16]	M, 20	Right side base of odontoid process	XR, CT, MRI, bone scintigraphy	Neck pain	Transoral endoscopic excision, C1–C2 transpedicle posterior fixation
Amendola, 2013 [17]	M, 23	Lower C2 endplate	XR, bone scintigraphy, CT	Neck pain, reduced ROM	Cervical antero-lateral endoscope-assisted biopsy and excision
Kaner, 2010 [3]	M,25	Left C2 lamina	CT, MRI	Neck pain	Laminectomy
Amirjamshidi, 2010 [14]	F, 32	Left aspect of the base of odontoid process	CT, MRI, bone scintigraphy	Neck pain, torticollis	Antero-lateral pre-vascular retropharyngeal approach and piecemeal resection; transoral approach and piecemeal resection
	M, 46	Odontoid process and left aspect of C2 body	CT, MRI, bone scintigraphy	Neck pain, torticollis	Anterior retropharyngeal approach and piecemeal resection
Arvin, 2009 [10]	F, 70	Left side of the C2 body	CT, MRI	Dysphagia, ear pressure	Transoral macroscopic excision
Gasbarrini, 2011 [18]	N/A	Body of C2	N/A	N/A	Anterior cervical endoscope-assisted excision, C2–C3 plating

C1, atlas; C2, axis; CT, computed tomography; F, female; M, male; MRI, magnetic resonance imaging; ROM, range of motion; XR, plain radiography.

## Data Availability

Not applicable.

## Supplementary Materials

The following supporting information can be downloaded at: https://doi.org/10.5281/zenodo.6684514, Video S1: Transnasal endoscopic approach for osteoid osteoma of the odontoid process in a child.
